# Inflammatory and homeostatic roles of eosinophil subpopulations in chronic rhinosinusitis with nasal polyp pathogenesis

**DOI:** 10.3389/fimmu.2025.1568541

**Published:** 2025-04-11

**Authors:** Kantapat Simmalee, Putthapoom Lumjiaktase, Theerasuk Kawamatawong, Amir Guemari, Valérian Dormoy, Joana Vitte

**Affiliations:** ^1^ Department of Pathology, Faculty of Medicine Ramathibodi Hospital, Mahidol University, Bangkok, Thailand; ^2^ Université de Reims Champagne-Ardenne, Institut national de la santé et de la recherche médicale (Inserm) Unité Mixte de Recherche (UMR)-S 1250 Pulmonary Pathologies and Cell Plasticity (P3Cell), Reims, France; ^3^ Division of Pulmonary and Critical Care Medicine, Department of Medicine, Faculty of Medicine Ramathibodi Hospital, Mahidol University, Bangkok, Thailand; ^4^ Immunology Laboratory, University Hospital of Reims, University of Reims Champagne-Ardenne, Reims, France

**Keywords:** chronic rhinosinusitis with nasal polyps, eosinophil, eosinophil subpopulations, inflammatory eosinophils, resident eosinophils

## Abstract

Chronic rhinosinusitis (CRS) with nasal polyps (CRSwNP) mainly expresses type-2 endotype, featuring eosinophils as a main player in the inflammatory process. Prolonged eosinophilia in the tissues of asthma and CRSwNP patients has been associated with structural changes, leading to fixed airflow obstruction in asthma and nasal polyposis in CRSwNP. This suggests that eosinophils may belong to different subgroups playing distinct roles in pathogenesis. Recent studies highlight the roles of inflammatory eosinophils (iEOS) in driving inflammation and tissue damage, whereas tissue-resident eosinophils (rEOS) maintain homeostasis and tissue repair in the airway. Therefore, understanding both roles of eosinophil subpopulations is crucial for better CRSwNP management, including enhancing the diagnosis accuracy, predicting recurrence, and optimizing treatment strategies.

## 1 Introduction

Chronic rhinosinusitis (CRS) with nasal polyps (CRSwNP) is an inflammatory nose disease with a complex mechanism of dysregulated immune responses, which significantly contributes to morbidity and reduced quality of life. According to a European Position Paper on Rhinosinusitis and Nasal polyps (EPOS) 2020, CRSwNP is characterized by the presence of two or more symptoms among nasal obstruction, facial pain/pressure, and hyposmia/anosmia more than or equal to 12 weeks with endoscopic based evidence of bilateral nasal polyposis (1).

The primary treatment for CRSwNP includes nasal irrigation, nasal spray, intranasal corticosteroids, and a short-course of oral corticosteroids. In refractory cases, functional endoscopic sinus surgery (FESS) is considered. Additionally, biological treatments are available for severe cases, such as dupilumab (monoclonal anti-IL-4Rα), mepolizumab (monoclonal anti-IL-5), and omalizumab (monoclonal anti-IgE). However, despite appropriate treatment, the need of systemic steroid use remains high, which is associated with adverse effects such as infections, diabetes, obesity, osteoporosis, cardiovascular disease, and cataracts. Furthermore, 40% of patients who underwent surgery experienced nasal polyp recurrence within 18 months, and 25% of surgery cases required revision procedures. This burden is more significant in those with comorbid asthma and high eosinophil levels (2–4).

The inflammation patterns underlie the heterogeneity in clinical manifestation and treatment responsiveness affecting disease management and outcomes. Identifying phenotypes (e.g., eosinophilic CRS, allergic fungal rhinosinusitis (AFRS), and non-eosinophilic CRS) and endotypes (e.g., type 2 and non-type 2) of CRSwNP improves the therapeutic options and clinical outcomes prediction after treatment. Endotypes have been widely utilized in clinical guidelines for planning personalized treatment. They are classified by the patterns of inflammatory drivers, including T helper (Th) cells and innate lymphoid cells (ILCs). The Type 2 endotype is characterized by increased T helper type 2 (Th2) cells and elevated levels of associated cytokines, which has a downstream influence on eosinophil numbers, specific IgE, and total IgE. In contrast, non-Type 2 endotypes are driven by increased T helper type 1 (Th1) and T helper type 17 (Th17) responses, resulting in elevated neutrophil levels. Most CRSwNP typically exhibit type 2 phenotype, primarily characterized by eosinophilic inflammation. Therefore, detecting abnormalities in eosinophils is crucial for effective management in this group (1). Currently, only a limited number of biomarkers, including peripheral eosinophilia, tissue eosinophilia count, and IgE, are available for type-2 endotype (5). This limitation highlights the need for a deeper understanding of the mechanisms driving CRSwNP pathobiology. Additionally, advance knowledge of eosinophils is essential to identify potential biomarkers to improve diagnostic, prognostic, and therapeutic strategies (5).

Eosinophils are remarkable players in the pathogenesis of CRSwNP, particularly in severe and refractory forms (6). Blood and tissue eosinophilia are reported in approximately 80% of all CRSwNP cases and correlate with poor disease control and a higher risk of recurrence after FESS (5). Recent studies have shown that eosinophils not only have inflammatory and destructive roles but also contribute to homeostasis and tissue repair (7–9). These data indicate the functional diversity within the eosinophil population, leading to the concept of subpopulations in the eosinophils and disease manifestation and outcomes. In this review, we summarize the current knowledge on eosinophil subpopulations in CRSwNP, their pathophysiologic role, and the potential ways of harnessing them to manage this eosinophilic disease.

## 2 Basic features of eosinophil homeostasis

**Figure 1:**
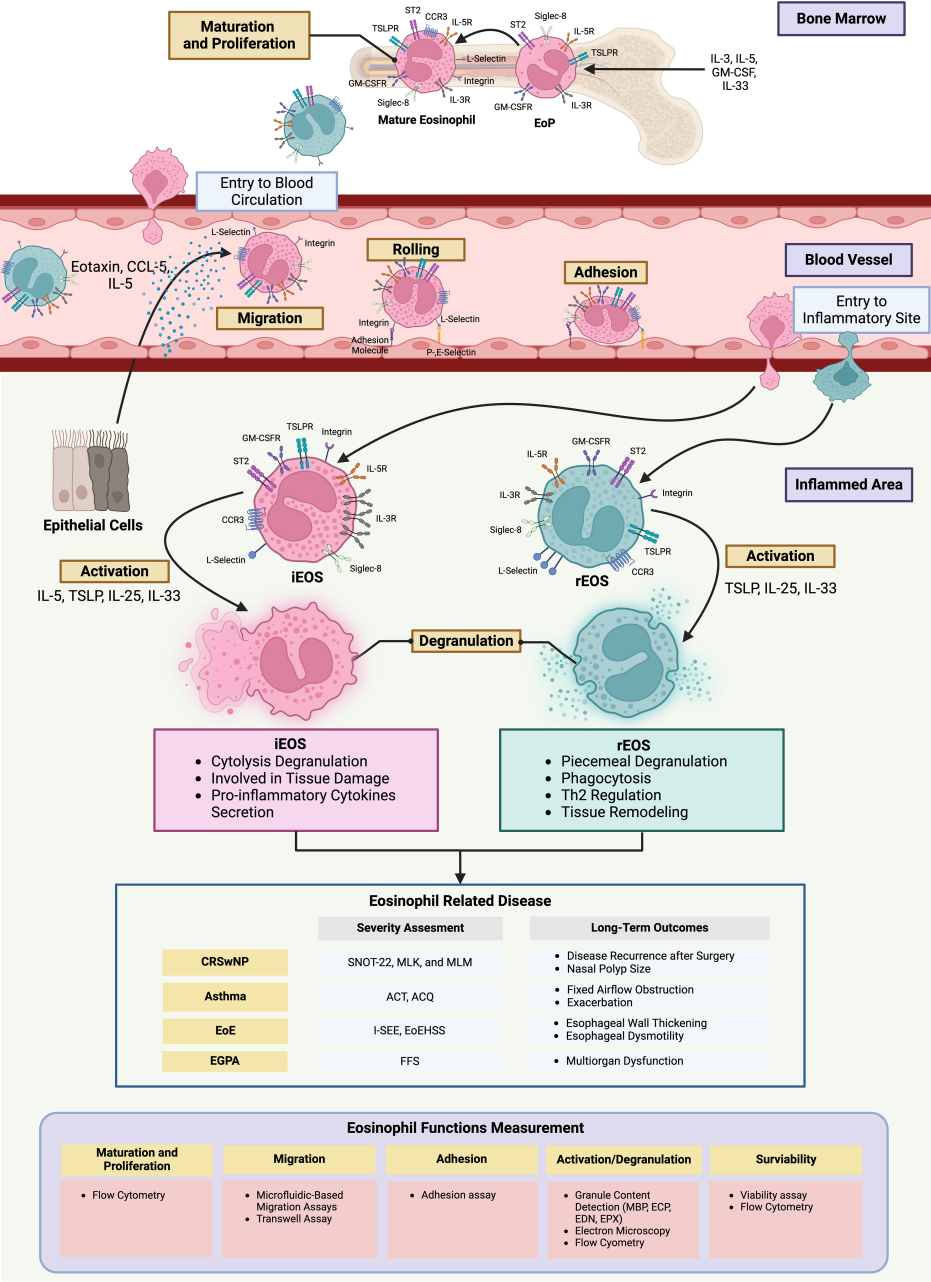
The different role of eosinophil subgroups in chronic inflammatory diseases The increase of pro-inflammatory cytokines from the inflamed areas induces the eosinophil progenitors to proliferate, mature, and differentiate into rEOS and iEOS before entering the bloodstream. Eosinophils are directed to the site of inflammation by chemokines. Their movement becomes slower and trapped by the elevated expression of adhesion molecules on epithelial cells before penetrating into the inflamed site. Within this microenvironment, eosinophils become activated and release cytokines and granules, contributing to pathogenesis and adverse outcomes in diseases such as CRSwNP, asthma, EoE, and EGPA. To create a strategy for managing these diseases, several technology facilitates the investigation of the differences in eosinophil function in individuals. Figure created with www.BioRender.com ACQ, Asthma Control Questionnaire; ACT, Asthma Control Test; EGPA, Eosinophilic granulomatosis with polyangiitis; EoE, Eosinophilic Esophagitis; EoEHSS, Eosinophilic Esophagitis Histologic Scoring System EoP, Eosinophil progenitors; FFS, Five-factor score; I-SEE, Index of Severity for Eosinophilic Esophagitis; iEOS, inflammatory eosinophil; MLK, Modified Lund Mackey; MLM, Modified Lund Kenedy; rEOS, resident eosinophil; SNOT-22, Sino-nasal Outcome Test-22.

Eosinophils originate in bone marrow before differentiation, proliferation, and activation through IL-3, GM-CSF, and IL-5 cytokine signals. They migrate from bloodstream to the inflamed tissue following a gradient of chemokines such as eotaxin, CCL-5, and the cytokine IL-5 (10). The high expression of adhesion molecules on epithelial cells at the inflamed area facilitates eosinophil attachment via L-selectins and integrins, inducing diapedesis into the tissue (11). Upon eosinophils arrive at the target organs, lipid mediators and cytokines in the surrounding milieu trigger their activation and degranulation (12) (**Figure 1**). Eosinophil granule proteins, such as major basic protein (MBP), eosinophil cationic protein (ECP), eosinophil peroxidase (EPO), and eosinophil-derived neurotoxin (EDN), exhibit cytotoxic effects able to neutralize microorganisms and parasites. However, their non-selective mechanism also causes severe damage to host tissues, potentially leading to the pathogenesis of various inflammatory diseases such as asthma, CRS, nasal polyps, and eosinophilic esophagitis (EoE) (8). Furthermore, cytokines released by eosinophils, such as IL-4, IL-5, and IL-13, enhance the inflammatory processes by activating and attracting other immune cells in the type-2 high pathway (12). Eosinophils may access lymph nodes from high endothelium venules and provide IL-4 to T cells undergoing activation and proliferation, thus sustaining the Th2 orientation of the immune response (13).

## 3 Subpopulations of eosinophils

Eosinophils generally survive in circulation for 3 to 18 hours and represent less than 5% of the total leukocyte count. However, large amounts of eosinophils are present in tissues such as adipose tissue, uterus, mammary glands, gastrointestinal (GI) tract, hearts, lungs, and thymus, with a half-life extending from 36 hours to 1 week (8, 10, 14, 15). Eosinophils in the nasal polyp tissue tend to live longer than 2 weeks because they express the antiapoptotic genes, such as *BCL2A1, BCL2L1, BCL3, BIRC2, BIRC3, TNFAIP3, PPP1R15A*, which inhibit the apoptosis pathway. Moreover, tissue eosinophils exhibit higher activation and prolonged survival, driven by IL-5 from the inflammatory microenvironment, compared with peripheral eosinophils. IL-5 sustains eosinophil survival by inhibiting apoptosis through the JAK-STAT pathway (6, 14, 16). Furthermore, it was shown that the eosinophil population dominates over other immune cells in the upper airway tissue of both those with or without CRS. The eosinophil proportion is higher in CRSwNP than in CRS without nasal polyps (CRSsNP) groups. Surprisingly, their proportion is higher in controls than in the CRSsNP groups. These data indicate that substantial eosinophil populations may play a role in inducing inflammation, while others may have different functions (17).

The specific population of resident EOS (rEOS) was recently identified in mice lung tissue by surface markers Siglec-F^int^CD62L^+^CD101^low^ without effect from IL-5 and located at the lamina propria. In contrast, an IL-5-dependent population of inflammatory EOS (iEOS) characterized by Siglec-F^hi^CD62L^-^CD101^hi^ is recruited during a house-dust-mite (HDM) treatment and is dense near the epithelial layer (8). Similar eosinophil subpopulations have been identified in the human pulmonary and nasal polyp tissues through the different expression of CD62L and IL-3R as Siglec-8^+^CD62L^+^IL3R^-^ cells for iEOS, and Siglec-8^+^CD62L^-^IL3R^+^ for rEOS (8, 18–20). Eosinophil density is high throughout the nasal lamina propria and the epithelial layer of CRSwNP and CRSsNP (17), however, the roles of the different eosinophil populations have not yet been elucidated.

The gene expression profiles of rEOS and iEOS reveal distinct roles in the immune response. rEOS are involved in homeostasis, tissue repair, antimicrobial defense, and decrease the Th2 response, while iEOS express several inflammation-associated genes (8). Electron microscopy evidenced structural differences between the two subsets, with rEOS exhibiting piecemeal degranulation (PMD), releasing a small number of selective granule packages over time, while iEOS displayed massive degranulation and no PMD (8). The degranulation observed with rEOS may relate to the regulatory immune response, immune cell recruitment to the inflamed site, antimicrobial defense, and tissue repair by secreting various proteins (8, 20). Conversely, iEOS cytolytic degranulation releases all the granules, which include MBP, ECP, EPO, and EDN, leading to tissue damage and prolonged inflammation. In the disease condition, the excessive cytokines released, such as IL-5 promote the survival and enhance the function of iEOS due to their IL-5-dependent, resulting in high population and hyperactivate of iEOS that may contribute to the pathogenesis of eosinophilic diseases (8). This is related to the observation in nasal sinus tissue from eosinophilic CRS, showing the degranulation pattern is half of PMD, and the rest shows cytolysis (21).

However, the roles of these subgroups are still controversial. The study on colitis found active eosinophils (A-EOS) in the epithelial layer of the colon have a function in homeostasis and bacteria elimination. This function is similar to rEOS, but rEOS is located in the lamina propria of tissue (22). Additionally, single-cell RNA sequencing (scRNA-seq) of eosinophils from nasal polyp of CRSwNP patients has identified four distinct expressions of subpopulations in chemokines (*CCL3*, *CCL4*, and *CXCL8*), proinflammatory cytokines (*INHBA)*, and growth factors (*ARL4C*). Each of these clusters requires different stimuli, such as IL-1β or IL-33, to express their specific cytokines, but the exact stimuli for each subpopulation are unclear (6). Overall, these findings indicate the existence of eosinophils with specific functions in homeostasis and inflammation within sinonasal tissue. The understanding of the specific roles of these subpopulations remains limited (**Figure 1**).

## 4 The imbalance of eosinophil subpopulations in CRSwNP

The imbalance between two subtypes of eosinophils might contribute to the increased severity of the diseases (19). However, evidence on the proportion of iEOS and rEOS in the CRSwNP is lacking. Data from asthmatic patients help us understand the roles of both subpopulations in the CRSwNP, as both diseases share pathogenic similarities. The patients with CRSwNP have an increased likelihood of comorbidity with asthma, suggesting that an imbalance between iEOS and rEOS might be present, as observed in the nasal polyp tissue from asthma patients (23). The percentage of iEOS was higher in the peripheral blood of asthma patients compared to healthy controls (10). Moreover, the percentage of iEOS is higher in blood samples of severe refractory asthma (18). In contrast, another study has reported a lower iEOS percentage in severe asthma compared to asthma and healthy control (19). This inconsistency may be due to the lack of data on absolute eosinophil count in each subgroup. The findings suggest that while the relative percentage appears lower, the actual iEOS number in severe asthma might be elevated compared to other groups (10, 19). On the other hand, the iEOS may have already infiltrated into the tissue when responding to chemokines released from the inflammation site (10, 19). This hypothesis is supported by data showing that the percentage of iEOS is higher in the nasal polyp tissue than in peripheral blood samples of asthma (18). Moreover, the percentage of iEOS is significantly decreased in the blood sample of asthma patients after challenges with the allergen (19). This evidence has shown that the iEOS might migrate to the inflamed area by IL-5, eotaxin, and alarmins [IL-25, IL-33, and thymic stromal lymphopoietin (TSLP)], while a small proportion of rEOS infiltrated into the airway tissue triggered by alarmins (10, 19). However, data on the velocity of both eosinophil subtypes in the bloodstream is lacking. iEOS may migrate faster to the inflamed site once attracted by chemokines, leading to the high percentage of iEOS observed in the nasal polyp of severe cases (18).

Although both eosinophil subpopulations in the peripheral blood of asthmatic patients are increased in adhesion, airway smooth muscle (ASM) migration, and extracellular matrix (ECM) production, rEOS perform better in all aspects. Nevertheless, iEOS shows more functional performance in reactive oxygen species (ROS) production than rEOS in asthma. These findings support the view that the over-functioning iEOS leads to inflammation in the airway tissue, whereas the rEOS plays a role in homeostasis and tissue repair. The hyperactivate of rEOS may contribute to the tissue thickening and remodeling in airway diseases. However, the ratio between these subpopulations that leads to imbalance is currently unknown (19, 20). Further research is needed to understand these populations in CRSwNP regarding clinical manifestations, prognosis, and treatment outcomes.

## 5 Future directions in clinical practice

Eosinophils have shown great potential in the clinical management of eosinophilic diseases such as asthma and CRSwNP. The characteristics of the patients, including type 2 and non-type 2 CRSwNP are associated with blood and tissue eosinophilia. The peripheral blood eosinophils of more than 500 cells/µl in CRSwNP patients is a promising biomarker predicting the 2-year post-operative nasal polyp recurrence (5). Moreover, blood or tissue eosinophil count evolution can guide targeted treatment strategies in clinical practice, such as dupilumab, mepolizumab, and omalizumab that are used to reduce inflammation in patients with severe symptoms and exacerbations (24).

The emerging role of iEOS and rEOS in airway diseases may offer new CRSwNP management strategies by enhancing our understanding of pathogenesis. Data from these subpopulations may improve the assessment of current and future severity of specific patient populations. For instance, the relative and absolute counts of activated iEOS and rEOS in a population could be more accurate severity biomarkers than the absolute count of blood or tissue eosinophils, especially in those who have high numbers of activated iEOS and low numbers of activated rEOS (12). The current systems for assessing disease severity rely mainly on clinical outcomes and quality of life, which include only a few biomarkers. Eosinophil subpopulations may also improve the severity assessment system in other eosinophil-associated diseases, such as asthma, eosinophilic granulomatosis with polyangiitis (EGPA), and EoE (25–28) (**Figure 1**).

A clinical study of dupilumab-treated CRSwNP also shows that blood eosinophil count remains stable, whereas total IgE levels decrease during the treatment (24). This may indicate that dupilumab reduces iEOS while increasing rEOS. On the other hand, dupilumab might reduce the activation of iEOS and simultaneously enhance the activation of rEOS. However, the effect of biological treatment on the eosinophil subpopulations remains unknown (29).

**Figure 2:**
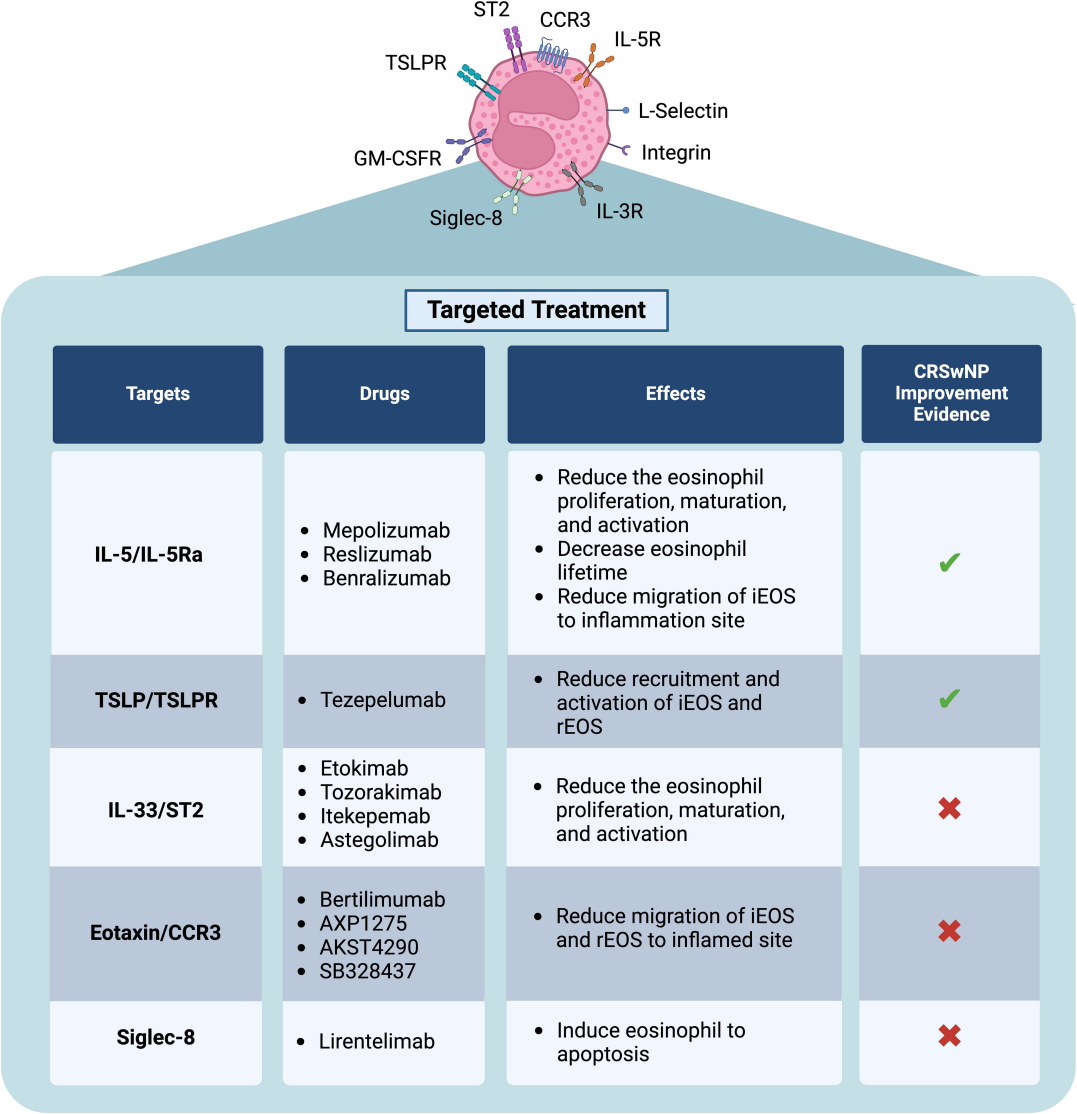
Novel treatment in CRSwNP specific to eosinophil function Current biological treatments approved for CRSwNP treatment. Some drugs may have a potential effect as therapeutics targeting eosinophil functions. Additionally, combining these drugs may enhance efficacy, particularly in patients who do not respond to current treatments. Figure created with www.BioRender.com .

These subpopulations may serve as potential therapeutic targets. The treatment strategies focusing on preventing iEOS from overactivation, which causes inflammation, and preventing rEOS from over-functioning, which causes airway thickening and nasal polyps from excessive tissue regeneration, is a promising issue. Alternatively, maintaining the stability of rEOS without overactivation may help regulate immune responses and sustain a healthy microenvironment in the sinonasal tissue (10). Therefore, targeting the eosinophil function is a key focus of emerging therapies. With the understanding of eosinophil behavior, targeted treatments can be developed. Therapeutic monoclonal antibodies directed to IL-5/IL-5Rα and IL-33 are expected to reduce the proliferation, differentiation, and maturation of eosinophils, leading to a decrease in the number of eosinophils released from the bone marrow (30, 31). Anti-IL-5 can also reduce the survival of eosinophils in blood and the migration of iEOS. Moreover, anti-IL-5, anti-TSLP, and anti-IL-33 also decrease the recruitment and activation of eosinophils (30, 32, 33). Clinical trials demonstrated that after treating CRSwNP patients with anti-IL-5/IL-5Rα, the eosinophil count in the blood decreased significantly, and clinical improvement was seen (30). The clinical trials with tezepelumab (monoclonal anti-TSLP) showed the potential for improving sinonasal symptoms of severe asthma comorbid with CRSwNP (32). However, some treatments, such as anti-IL-33, did not significantly reduce the Sino-Nasal Outcome Test-22 (SNOT-22) score, nasal polyp score (NPS), or eosinophil count, indicating insufficient therapeutic efficacy (33). In such cases, drug combinations could be an alternative option. For example, anti-eotaxin and anti-CCR3, which may reduce the migration signal of eosinophil, could be used in combination with anti-Siglec-8, which promotes eosinophil apoptosis (34–37). Currently, however, there are no data available on the effectiveness of these drugs or corticosteroids alone and the combined therapies on the iEOS and rEOS. Further studies on this topic could indeed provide critical insights for tailored therapeutic approaches in CRSwNP (29) (**Figure 2**).

Furthermore, integrating iEOS and rEOS into prognostic models could improve the prediction of the treatment response and recurrence events. Recently, the prognosis models demonstrated the personalized strategies of patient care. A study combining inflammatory cytokine profiles with clinical data successfully developed models to predict polyp recurrence, achieving an area under the curve of 0.89 (23). Although they do not include the eosinophil population data, the results highlighted potential biomarkers associated with eosinophils, such as ECP and IL-5, for predicting polyp recurrence. Integrating the subpopulations of eosinophils could enhance the accuracy of predictive models for nasal polyp recurrence. Additionally, these subpopulations may create the models that help us to classify CRSwNP patients based on their endotypes to identify treatable traits and develop potential treatment strategies, such as immunotherapy (23). This also benefits other eosinophil-related diseases in predicting long-term outcomes by using individual profiles. For instance, it can help to predict the fixed airway obstruction and exacerbation events in asthma patients, esophageal thickening or dysmotility of EoE, and the organ multifunction in EGPA, which are caused by eosinophil function. These diseases now have a targeted treatment specific to eosinophils. Integrating individual eosinophil-related data could help clinicians design treatment strategies to prevent adverse outcomes in the future (38–40) (**Figure 1**).

Several techniques improved the capture of eosinophil function details. For example, flow cytometry is used to evaluate the maturation and differentiation of eosinophils by quantifying the proportion of iEOS and rEOS (19). The microfluid-based migration assay and the transwell assay can evaluate the migration of eosinophil subpopulations (41). The adhesion assay is used to detect the percentage of adhesion eosinophil subpopulations by simulating the inflamed environment (19). iEOS and rEOS activation and degranulation can be assessed through multiple methods, including surface protein via the flow cytometry, secreted granule content using the ELSIA test, and degranulation patterns observed under electron microscopy (19, 21). Additionally, the survival of iEOS and rEOS in blood and tissue can be evaluated by assessing eosinophil viability after culture in an inflammatory environment. This can be done through various methods, such as annexin V detection via a flow cytometry-based assay or colorimetric assays (e.g., MTT assay) (19). These can also be used to detect the individual functions in iEOS and rEOS to create personalized management (**Figure 1**).

Collectively, the concepts of iEOS and rEOS have not yet been completely elucidated and well- standardized. In addition to these two populations, there are still other subpopulations and functions in CRSwNP awaiting description, as suggested by the scRNA-seq (6).

## 6 Conclusion

iEOS and rEOS play a key role in inflammation and homeostasis in the airway tissue. To date, the pathogenesis mechanism and roles of eosinophil subpopulations in CRSwNP are not well established. Here, we reviewed and summarized the current data on eosinophils associated with airway diseases, underlining the need for further experimental investigation of their contribution to the pathogenesis of CRSwNP, in order to harness new potential therapeutic targets and biomarkers for clinical implementation.
